# Firearm Possession Rates in Home Countries and Firearm Suicide Rates Among US- and Foreign-Born Suicide Decedents in the United States: Analysis of Combined Data from the National Violent Death Reporting System and the Small Arms Survey

**DOI:** 10.2196/44211

**Published:** 2023-09-29

**Authors:** In Han Song, Jin Hyuk Lee, Jee Soo Shin

**Affiliations:** 1 ICONS Convergence Academy Yonsei University Seoul Republic of Korea; 2 Graduate School of Social Welfare Yonsei University Seoul Republic of Korea; 3 Faculty of Medicine Vilnius University Vilnius Lithuania; 4 Department of Social Welfare Ewha Womans University Seoul Republic of Korea; 5 Interdisciplinary Program of Social Welfare Policy The Graduate School Yonsei University Seoul Republic of Korea

**Keywords:** firearm suicide, US born, foreign born, means of suicide, firearm possession rate, suicide decedents

## Abstract

**Background:**

Suicide by firearms is a serious public health issue in the United States. However, little research has been conducted on the relationship between cultural backgrounds and suicide by firearms, specifically in those born and raised in the United States compared to those who have immigrated to the United States.

**Objective:**

To better understand the relationship between cultural backgrounds and suicide, this study aimed to examine firearm suicide rates among US- and foreign-born suicide decedents based on the firearm possession rate in the decedent’s home country.

**Methods:**

Multivariate logistic regression was performed to analyze data of 28,895 suicide decedents from 37 states obtained from the 2017 National Violent Death Reporting System data set. The firearm possession rate in the home countries of foreign-born suicide decedents was obtained from the 2017 Small Arms Survey.

**Results:**

The firearm suicide rate was about twice as high among US-born suicide decedents compared to their foreign-born counterparts. Meanwhile, suicide by hanging was about 75% higher among foreign-born compared to US-born suicide decedents. Those from countries with a low-to-medium firearm possession rate were significantly less likely to use firearms compared to US-born suicide decedents (adjusted odds ratio [AOR]=0.45, 95% CI 0.31-0.65, and AOR=0.46, 95% CI 0.39-0.53, respectively). Meanwhile, firearm suicide rates were not different between US- and foreign-born suicide decedents from countries with a similarly high firearm possession rate.

**Conclusions:**

The results suggest that there is an association between using firearms as a means of suicide and the firearm possession rate in the decedent’s home country. Suicide by firearms in the United States needs to be understood in the sociocultural context related to firearm possession.

## Introduction

Currently, suicide by firearms is a serious public health issue in the United States. According to the annual mortality statistics of the Centers for Disease Control and Prevention (CDC) [1], about 100 people die by suicide every day in the United States, with around 50% of them using firearms, 28.6% using poison, and 12.9% using hanging as a means of suicide. Firearm suicide rates in the United States have steadily increased over the past few years [2,3]. The firearm possession rate is defined as an estimate of civilian firearms per 100 individuals in a country [4]. States, regions, and countries with higher firearm possession rates are associated with higher firearm suicide rates [5]. According to Miller et al [6] and Stack [7], firearm possession rates are positively correlated with firearm suicide rates in the United States. In fact, the most common means of suicide in the United States is death by firearms. Other countries with high firearm possession rates, such as Uruguay and Montenegro, have also reported high of suicide by firearms [4,8]. Meanwhile, in countries that do not have high firearm possession rates, such as Asian or Nordic countries, poisoning by pesticides is the most common means of suicide. Similarly, suicide by hanging is the most common means of suicide in Eastern Europe [9]. One evidence-based suicide prevention strategy is to reduce the social acceptance and availability of lethal means for those at risk of suicide [10,11]. Investigating the context around suicide, such as accessibility to specific means, can provide important information when devising suicide prevention interventions [12]. This is because restricting the means of suicide is known to be effective in preventing suicides [13,14].

According to Wong et al [15], the social acceptance and availability of the means of suicide are associated with which means of suicide are more likely to be chosen. In this study, *social acceptance* refers to the cultural beliefs surrounding firearms, familiarity with firearms, the normality of firearm possession, and the role that firearms play in daily life. These factors may influence an individual’s own perceptions about firearms and increase or decrease the likelihood of their owning their own personal firearm [16]. Meanwhile, *availability* in this study is defined as the widespread availability of and accessibility to firearms. Using firearms as the most common means of suicide is associated with the high household firearm possession rate in the United States [17]. However, compared to the volume of research on the availability of firearms and firearm suicide rates, little research has been conducted to understand the relationship between the social acceptance of firearms and firearm suicide rates. The United States is a nation made up of immigrants from many diverse countries. Because of this, it is important to be cognizant of different demographics and their relationships and associations with firearms and, as a result, the likelihood to die by firearm suicide. According to Liu et al [18], US-born individuals have a significantly higher firearm suicide rate, while foreign-born individuals have a significantly higher rate of suicide by hanging, suffocation, jumping from heights, and sharp instruments. Moreover, it is suggested that immigrants are influenced by the culture surrounding firearms in their home countries [19]. Assuming that there is an association between the cultural familiarity with and social acceptance of firearms and the probability of an individual using a firearm as a means of suicide, this cultural distinction may be important in analyzing the difference between US- and foreign-born suicide decedents. Therefore, in this research, we compiled information about firearm possession rates in the home countries of immigrant populations in order to analyze the relationship between firearm familiarity and suicide behaviors of choosing a means of suicide. Despite the critical importance of identifying factors that may impact suicidal behaviors, to the best of our knowledge, little research has been conducted on the firearm possession rate by home country to understand the relationship between cultural/ethnic backgrounds and the means of suicide. Thus, our study aimed to better understand the cultural differences around firearms and how these may impact the likelihood of an individual dying by firearm suicide.

With the goal to determine the relationship between cultural familiarity and the means of suicide, we compared the means of suicide between US- and foreign-born suicide decedents. Focusing on suicides by firearms, in particular, we divided foreign-born individuals by the firearm possession rate in their home countries and compared them with US-born suicide decedents in order to understand the connection between the gun culture of the home country and the familiarity with firearms as a means of suicide. Our findings can serve as baseline data for devising suicide prevention strategies that focus on the restriction of access to various means of suicide, consider sociocultural backgrounds related to specific means, and customize the strategies to the sociocultural backgrounds of target groups.

## Methods

### Study Design

The National Violent Death Reporting System (NVDRS) is a state-based active surveillance system that provides a detailed account of violent deaths that occur in participating states. The NVDRS provides abundant data on the details of violent deaths in the United States, and it is the first national system to collect comprehensive information about suicides [20]. Moreover, the NVDRS is the nation’s only reporting system for complex events, such as homicide-suicide and mass killings, and offers far richer information about all other violent deaths compared to other data sources [21]. With support from the CDC, the system collects data from multiple sources, including death certificates, police reports, medical examiner/coroner reports, and, sometimes, crime lab information [22].

This study used restricted data of decedents provided by the 2017 NVDRS and examined only deaths resulting from suicide. In 2017, 37 states (Alaska, Arizona, California, Colorado, Connecticut, Delaware, District of Columbia, Georgia, Illinois, Indiana, Iowa, Kansas, Kentucky, Maine, Maryland, Massachusetts, Michigan, Minnesota, Nevada, New Hampshire, New Jersey, New Mexico, New York, North Carolina, Ohio, Oklahoma, Oregon, Pennsylvania, Puerto Rico, Rhode Island, South Carolina, Utah, Vermont, Virginia, Washington, West Virginia, and Wisconsin) contributed data to the NVDRS. The 2017 NVDRS data include decedents who died from violent deaths in 2017. Specifically, the data include 33,257 individuals who died by suicide; data on the means of suicide and the home country are missing for 4362 (13.1%) decedents. Our final sample consisted of 28,895 decedents after removing individuals with missing data. So, this study used complete case analysis (CCA) to deal with missing values.

### Measurements

The means of suicide were used as the outcome variable in this study. In the NVDRS data, the means of suicide include firearms, hanging or suffocation, poisoning, jumping from a height, sharp instruments, drowning, and other. However, this study used firearms, hanging or suffocation, and poisoning as the main outcome variables. Nativity, the independent variable, was divided into US- and foreign-born suicide decedents. People born in the United States, Puerto Rico, and a US Island Area (Guam, the Commonwealth of the Northern Mariana Islands, or the US Virgin Islands) were defined as US born, and those born outside the United States were defined as foreign born [23].

The firearm possession rate by home country was obtained from the estimate of civilian firearms per 100 individuals per country provided by the 2017 Small Arms Survey (SAS) [4]. The SAS is an independent research project located at the Graduate Institute of International and Development Studies in Geneva, Switzerland. The SAS uses data from various sources, such as official documents, studies, questionnaires, public opinion surveys, news reports, and expert correspondence, to estimate civilian firearm ownership. It systematically integrates these data to generate total estimates for each country, while excluding extreme high and low numbers as outliers, when feasible. This data set is used because it provides information about firearm possession rates for most countries. We matched the information about firearm possession rates to the home countries of the foreign-born individuals. A country with a firearm possession rate of <1 SD from the mean value was coded as “low,” of >1 SD from the mean value was coded as “high,” and of 1 SD from the mean value was coded as “medium.” Sociodemographic variables included race, age, gender, region, education, and marital status.

### Ethical Considerations

This study was exempt from human subject research ethics by the Institutional Review Board of Yonsei University (7001988-202010-HR-1013-01E) due to secondary analysis of research data. The data were derived from routine injury mortality surveillance. The NVDRS is incident based and links all victims and alleged perpetrators (suspects) associated with a given incident in 1 record. To fully characterize incidents, states collect information about deaths from numerous data sources. These sources include death certificates, coroner/medical examiner reports, and law enforcement reports [22].

### Statistical Analysis

A descriptive analysis was conducted on the general characteristics of suicide decedents. Rates per 100,000 people (by nativity, race, sex, and age group) were calculated using the census data of the 2017 American Community Survey. Logistic regression was performed to analyze the association between nativity and the means of suicide. The independent variables of this study were nativity (US born/foreign born) and the firearm possession rate in the home country. The dependent variable was the choice of a means of suicide (firearm, hanging, and poisoning). Other means of suicide were excluded from the logistic analysis in order to explain the 3 major means of suicide.

First, logistic regression was performed to compare the correlation between sociodemographic variables and the means of suicide. Second, we analyzed the association between nativity and the means of suicide after adjusting for gender, age, place of residence, education, and marital status. Next, we analyzed the association between the firearm possession rate in the home country and the means of suicide. We used the US-born suicide decedents as a reference group for the 2 independent variables and examined how the choice of a means of suicide for the 2 independent variables is different compared to the US-born suicide decedents. The adjusted odds ratio (AOR) and 95% CI were presented, and statistical significance at the α level of .05 was indicated by 95% CIs that did not include 1. All analyses were performed using STATA, version 13.

## Results

### Demographic Characteristics of Suicide Decedents

Tables 1 and 2 show the demographic characteristics of suicide decedents in 37 states presented by the 2017 NVDRS. Of the total 28,895 deaths by suicide, the firearm suicide rate was 5.9 per 100,000 people, the hanging suicide rate was 3.7 per 100,000 people, the poisoning suicide rate was 1.6 per 100,000 people, and the rate of suicide by other means was 1.0 per 100,000 people. Firearm suicide rates were higher than hanging and poisoning suicide rates among US-born, White, Black, male, and 18-year-old or older decedents. The hanging suicide rates were higher compared to firearm and poisoning suicide rates among were foreign born; belonged to Hispanic, Asian/Pacific Islander, or other ethnic groups; and were less than 18 years old. The poisoning suicide rates were slightly higher than firearm and hanging suicide rates among women.

**Table 1:** Demographics of suicide decedents by means of suicide: 2017 NVDRS^a^.

Characteristics			Decedents (N=28,895), n (%)		Firearms (n=14,041, 48.6%), n (%)		Hanging (n=8679, 30.0%), n (%)		Poisoning (n=3900, 13.5%), n (%)		Other^b^ (n=2275, 7.9%), n (%)
**Nativity**											
	US born	27,252 (94.3)		13,561 (96.6)		7922 (91.3)		3716 (95.3)		2053 (90.2)	
	Foreign born	1643 (5.7)		480 (3.4)		757 (8.7)		184 (4.7)		222 (9.8)	
**Race**											
	White	23,681 (82.0)		12,062 (85.9)		6534 (75.3)		3414 (87.5)		1671 (73.5)	
	Black	1812 (6.3)		874 (6.2)		547 (6.3)		183 (4.7)		208 (9.1)	
	Hispanic	2120 (7.3)		668 (4.8)		1024 (11.8)		186 (4.8)		242 (10.6)	
	Asian/Pacific Islander	566 (2.0)		148 (1.1)		265 (3.1)		52 (1.3)		101 (4.4)	
	Other	716 (2.5)		289 (2.1)		309 (3.6)		65 (1.7)		53 (2.3)	
**Sex**											
	Male	22,454 (77.7)		12,164 (86.6)		6723 (77.5)		1890 (48.5)		1677 (73.7)	
	Female	6441 (22.3)		1877 (13.4)		1956 (22.5)		2010 (51.5)		598 (26.3)	
**Age (years)**											
	<18	1092 (3.8)		415 (3.0)		551 (6.3)		72 (1.8)		54 (2.4)	
	18-24	3149 (10.9)		1498 (10.7)		1159 (13.4)		224 (5.7)		268 (11.8)	
	25-44	9443 (32.7)		3922 (27.9)		3668 (42.3)		1101 (28.2)		752 (33.1)	
	45-64	10,125 (35.0)		4750 (33.8)		2638 (30.4)		1848 (47.4)		889 (39.1)	
	≥65	5086 (17.6)		3456 (24.6)		663 (7.6)		655 (16.8)		312 (13.7)	

^a^NVDRS: National Violent Death Reporting System.

^b^“Other” includes jumping from heights, sharp instruments, drowning, and other means.

**Table 2:** Rate of suicide per 100,000 people by means of suicide: 2017 NVDRS^a^.

Characteristics			Decedents, rate/100,000		Firearms, rate/100,000		Hanging, rate/100,000	Poisoning, rate/100,000		Other^b^, rate/100,000
Total			12.2		5.9		3.7	1.6		1.0
**Nativity**										
	US born	13.3		6.6		3.9		1.8	1.0	
	Foreign born	5.0		1.5		2.3		0.6	0.7	
**Race**										
	White	15.8		8.1		4.4		2.3	1.1	
	Black	6.8		3.3		2.0		0.7	0.8	
	Hispanic	5.5		1.7		2.6		0.5	0.6	
	Asian/Pacific Islander	3.8		1.0		1.8		0.4	0.7	
	Other	9.0		3.6		3.9		0.8	0.7	
**Sex**										
	Male	19.2		10.4		5.7		1.6	1.4	
	Female	5.3		1.6		1.6		1.7	0.5	
**Age (years)**										
	<18	2.0		0.8		1.0		0.1	0.1	
	18-24	13.6		6.5		5.0		1.0	1.2	
	25-44	15.0		6.2		5.8		1.8	1.2	
	45-64	16.2		7.6		4.2		2.9	1.4	
	≥65	14.5		9.8		1.9		1.9	0.9	

^a^NVDRS: National Violent Death Reporting System.

^b^“Other” includes jumping from heights, sharp instruments, drowning, and other means.

First, we examined the correlation between demographic variables and the choice of a means of suicide. The results are presented in Tables 3-5. Compared to Whites, Blacks were more likely to use firearms but less likely to use hanging or poisoning as a means of suicide. Compared to Whites, Hispanic, Asian/Pacific Islander, and other ethnic minorities were more likely to use hanging or poisoning and less likely to select firearms as a means of suicide. Older people were more likely to select firearms or poisoning and less likely to choose hanging as a means of suicide. Compared to men, women selected poisoning more than firearms. Urban dwellers were less likely to choose firearms and more likely to use hanging or poisoning. The higher the education level, the fewer the number of suicides by hanging and the more the number of suicides by poisoning. Those with spouses used firearms but not as much as poisoning as a means of suicide.

**Figure 1:**
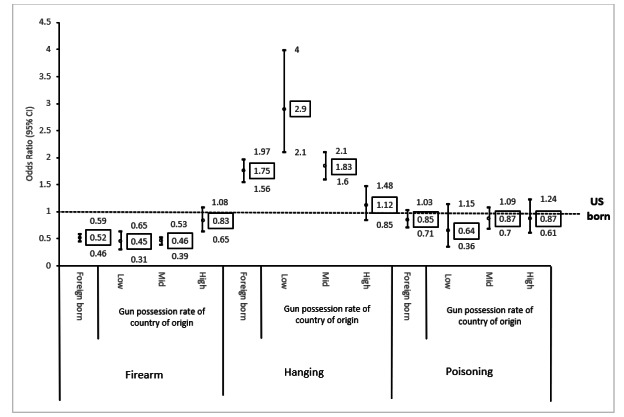
Means of suicide among suicide decedents from the United States and other countries with different levels of the firearm possession rate: 2017 National Violent Death Reporting System.

Next, we tested the association between nativity and the means of suicide after adjusting for demographic variables. The results are shown in Tables 3-5 and Figure 1. We compared the choice of a means of suicide between US- and foreign-born suicide decedents. Compared to US-born suicide decedents, their foreign-born counterparts were less likely to use firearms and more likely to select hanging as a means of suicide. Next, we compared the means of suicide between US- and foreign-born suicide decedents based on the firearm possession rate in the home countries of the latter. People who immigrated from countries with a low firearm possession rate selected firearms more than they did hanging as a means of suicide compared to US-born suicide decedents. People who were from countries with a medium firearm possession rate chose firearms less than they did hanging as a means of suicide compared to US-born suicide decedents. However, the difference between foreign-born suicide decedents from countries with a high firearm possession rate and their US-born counterparts was not statistically significant.

**Table 3:** Statistics of suicide by firearms among suicide decedents from the United States and other countries with different levels of the firearm possession rate: 2017 NVDRS^a^.

Characteristics			Model 1			Model 2	
			AOR^b^ (95% CI)		*P* value	AOR (95% CI)	*P* value
Foreign born (reference: US born)			0.52 (0.46-0.59)		<.001	N/A^c^	N/A
**Firearm possession rate in the home country (reference: US born)**							
	Low	N/A		N/A		0.45 (0.31-0.65)	<.001
	Medium	N/A		N/A		0.46 (0.39-0.53)	<.001
	High	N/A		N/A		0.83 (0.65-1.08)	.162
**Race (reference: White)**							
	Black	1.20 (1.09-1.33)		<.001		1.21 (1.1-1.35)	<.001
	Hispanic	0.58 (0.52-0.64)		<.001		0.60 (0.54-0.66)	<.001
	Asian/Pacific Islander	0.62 (0.51-0.77)		<.001		0.67 (0.54-0.83)	<.001
	Other	0.77 (0.66-0.91)		.002^d^		0.78 (0.66-0.91)	.002^d^
Age			1.01 (1.01-1.02)		<.001	1.01 (1.01-1.02)	<.001
Female			0.35 (0.33-0.37)		<.001	0.35 (0.33-0.37)	<.001
Urban dweller			0.65 (0.61-0.69)		<.001	0.65 (0.61-0.69)	<.001
Education level			0.99 (0.97-1.01)		.160	0.99 (0.97-1.01)	.144
Married			1.40 (1.32-1.48)		<.001	1.40 (1.33-1.48)	<.001

^a^NVDRS: National Violent Death Reporting System.

^b^AOR: adjusted odds ratio.

^c^N/A: not applicable.

^d^*P*<.01.

**Table 4:** Statistics of suicide by hanging among suicide decedents from the United States and other countries with different levels of the firearm possession rate: 2017 NVDRS^a^.

Characteristics			Model 1			Model 2	
			AOR^b^ (95% CI)		*P* value	AOR (95% CI)	*P* value
Foreign born (reference: US born)			1.75 (1.56-1.97)		<.001	N/A^c^	N/A
**Firearm possession rate in the home country (reference: US born)**							
	Low	N/A		N/A		2.90 (2.10-4.00)	<.001
	Medium	N/A		N/A		1.83 (1.60-2.10)	<.001
	High	N/A		N/A		1.12 (0.85-1.48)	.435
**Race (reference: White)**							
	Black	0.80 (0.71-0.89)		<.001		0.79 (0.70-0.88)	<.001
	Hispanic	1.62 (1.47-1.79)		<.001		1.60 (1.45-1.77)	<.001
	Asian/Pacific Islander	1.36 (1.12-1.64)		.002^d^		1.21 (0.99-1.47)	.062
	Other	1.45 (1.24-1.70)		<.001		1.44 (1.23-1.68)	<.001
Age			0.98 (0.97-0.98)		<.001	0.98 (0.97-0.98)	<.001
Female			1.01 (0.95-1.08)		.718	1.01 (0.95-1.08)	.108
Urban dweller			1.27 (1.18-1.36)		<.001	1.27 (1.18-1.36)	<.001
Education level			0.95 (0.93-0.97)		<.001	0.95 (0.93-0.97)	<.001
Married			0.95 (0.89-1.01)		.120	0.95 (0.89-1.01)	.108

^a^NVDRS: National Violent Death Reporting System.

^b^AOR: adjusted odds ratio.

^c^N/A: not applicable.

^d^*P*<.01.

**Table 5:** Statistics of suicide by poisoning among suicide decedents from the United States and other countries with different levels of the firearm possession rate: 2017 NVDRS^a^.

Characteristics			Model 1			Model 2	
			AOR^b^ (95% CI)		*P* value	AOR (95% CI)	*P* value
Foreign born (reference: US born)			0.85 (0.71-1.03)		.095	N/A^c^	N/A
**Firearm possession rate** **in** **the home country (reference: US born)**							
	Low	N/A		N/A		0.64 (0.36-1.15)	.135
	Medium	N/A		N/A		0.87 (0.70-1.09)	.223
	High	N/A		N/A		0.87 (0.61-1.24)	.434
**Race (reference: White)**							
	Black	0.69 (0.58-0.82)		<.001		0.69 (0.59-0.82)	<.001
	Hispanic	0.69 (0.58-0.81)		<.001		0.68 (0.58-0.81)	<.001
	Asian/Pacific Islander	0.55 (0.39-0.75)		<.001		0.57 (0.41-0.80)	.001^d^
	Other	0.67 (0.51-0.88)		.004^d^		0.67 (0.51-0.88)	.004^d^
Age			1.01 (1.01-1.02)		<.001	1.01 (1.01-1.02)	<.001
Female			5.05 (4.69-5.43)		<.001	5.05 (4.69-5.44)	<.001
Urban dweller			1.32 (1.20-1.46)		<.001	1.32 (1.20-1.46)	<.001
Education level			1.05 (1.02-1.07)		<.001	1.05 (1.02-1.07)	<.001
Married			0.67 (0.62-0.73)		<.001	0.67 (0.62-0.73)	<.001

^a^NVDRS: National Violent Death Reporting System.

^b^AOR: adjusted odds ratio.

^c^N/A: not applicable.

^d^*P*<.01.

## Discussion

### Principal Findings

This research examined the data of 28,895 suicide decedents from 37 states included in the 2017 NVDRS with the goal of comparing the means of suicide between US- and foreign-born American suicide decedents and analyzing whether these differences could be explained by the culture surrounding firearms in their home countries, specifically firearm possession rates that illustrate a cultural familiarity with firearms. We found statistically significant differences in the means of suicide between US- and foreign-born individuals. The firearm suicide rate was about twice as high among US-born suicide decedents compared to their foreign-born counterparts in 2017, while the hanging suicide rate was about 75% higher among foreign-born suicide decedents compared to their US-born counterparts. We divided the home countries of the foreign-born suicide decedents into 3 groups by their firearm possession rate (low, medium, and high) and compared their means of suicide with those of the US-born suicide decedents. Those from countries with low and medium firearm possession rates were significantly less likely to use firearms compared to the US-born suicide decedents (AOR=0.45, 95% CI 0.31-0.65, and AOR=0.46, 95% CI 0.39-0.53, respectively). However, there was no statistical difference in the use of firearms between foreign-born suicide decedents from countries with a high firearm possession rate and their US-born counterparts. Foreign-born individuals from countries with a low or medium firearm possession rate were significantly more likely to use hanging compared to a firearm (AOR=2.90, 95% CI2.10-4.00, and AOR=1.83, 95% CI 1.60-2.10, respectively). In the case of suicide by poisoning, there was no statistical difference between US-born and all foreign-born suicide decedents. Compared to US-born individuals, immigrants from countries with a high firearm possession rate had no significant difference in firearm suicide rates and immigrants from countries with a low firearm possession rate had a significantly lower firearm suicide rate.

The findings of this research are in line with those of Liu et al [18], who found that US-born suicide decedents have a greater firearm suicide rate and a lower hanging suicide rate compared to their foreign-born counterparts and that there is no significant difference in the poisoning suicide rate between US- and foreign-born Americans. Furthermore, our results were confirmed by Wong et al [15], who found that US-born individuals have a significantly greater firearm suicide rate, a lower hanging suicide rate, and no significant difference in the poisoning suicide rate compared to foreign-born Asian/Pacific Islander Americans. In addition, this research compared cultural differences regarding firearms for the various home countries of immigrants residing in the United States who died by suicide. This confirms that there is an association between the firearm possession rate in the home country and the prevalence of various means of suicide. Furthermore, sociocultural familiarity with firearms is associated with a greater likelihood to die by firearm suicide [15]. Those born in a country with a high firearm possession rate are more likely to be exposed to and familiar with firearms, becoming more accepting of guns as a means of suicide, compared to those born in a country with a low firearm possession rate. This argument is supported by a number of studies: Conner et al [24] concluded that the means of suicide is influenced by the culture and gun policy of the home country, Kaplan and Geling [25] described the cultural origin of firearm suicide, Price et al [26] argued that firearm suicide is related to the gun laws of the country where an individual lives, Miller et al [27] supported that both firearm suicide and total suicide rates are associated with state and regional gun prevalence, and Saunders et al [19] suggested that immigrants are influenced by the firearm possession culture of their home country.

Considering that cultural perceptions of firearms can make certain means of suicide appear more manageable [28], it is understood that firearms are most commonly used for suicide in the United States because of their availability [29]. In contrast, hanging is the most common means of suicide in several Asian countries, including Japan, South Korea, and the Philippines [17]. In addition, this research found that the rate of suicide by hanging is high among immigrants from countries with a low firearm possession rate compared to US firearm possession rates.

It is necessary to devise suicide prevention strategies that focus on restricting access to dangerous items for at-risk individuals and that also consider the sociocultural implications of this topic. This is important as certain cultural backgrounds may be more sensitive to the exposure of the means of suicide. Suicide prevention efforts targeting war veterans include the establishment of suicide prevention campaigns and laws, such as the Joshua Omvig Veterans Suicide Prevention Act. In addition, the Veterans Agency and the Department of Defense provide free gun-safe storage to veterans at risk of dying by firearm suicide [30]. This measure is expected to be beneficial, given prior research that has proven the efficacy of proper gun safety and storage in reducing suicides among veterans [31]. Furthermore, it is imperative that suicide prevention campaigns be customized to best fit the needs of various sociocultural backgrounds. The World Health Organization’s National Strategy of Suicide Prevention notes the importance of restricting access to the means of suicide but fails to consider the way in which gun violence disproportionately affects certain sociocultural and ethnic backgrounds. There should be further research on this intersection between suicide rates and various sociocultural and ethnic groups in order to develop more effective suicide prevention campaigns. Wong et al [15] suggest that given the prevalence of suicide by firearms among the US-born population and suicide by hanging among immigrants, public health professionals should advise individuals with suicidal thoughts to remove potentially dangerous items from their environments. Based on our results, there seems to be an association between the culture surrounding firearms that an individual grew up in and the likelihood of them choosing suicide by firearms as opposed to other means. Therefore, public health professionals may need to follow culturally sensitive approaches to establish suicide prevention policies and strategies by reflecting on the cultural backgrounds of the population. In addition, when devising gun safety campaigns, states with high proportions of foreign-born individuals should consider their cultural backgrounds. Foreign-born individuals from countries with high firearm possession rates and US-born individuals are priority populations. In the case of both US- and foreign-born individuals from cultures highly familiar with firearms at risk of suicide, reducing access to lethal means is an evidence-based suicide prevention initiative [10,11]. Ideally, firearm accessibility should be limited in the United States to suppress the innate and widespread culture of firearm possession that has arisen in the country and has led to increased gun violence throughout the country. To alleviate this, federal gun policies must be enforced to reduce gun violence in the United States, including suicide by firearms. Such policies could include banning guns in public spaces, requiring a stricter process prior to buying a gun, banning high-capacity magazines and assault rifles, enforcing that all gun owners must store their guns in an approved gun storage unit, and requiring gun safety courses for everyone who wishes to own a gun. These measures will evidently reduce gun violence and particularly suicide by firearms [22]. The study is meaningful because it shows empirical results that the cultural background is related to suicide by firearms. Although the causal relationship is limited, the significant correlation means that cultural perspectives must be considered in planning firearm suicide prevention policies and interventions in a multicultural society, such as the United States.

### Limitations

There are several limitations of this research. First, the NVDRS data used in this study had 13% missing values due to difficulties in obtaining some sensitive information about suicide decedents. To deal with the missing values, this study used CCA. This is because compared to multiple imputation, CCA has been reported to provide unbiased estimates in cases where the missing percentage is less than 20%, and this missing percentage refers to missing completed at random (MCAR) or missing completed random (MAR) [32]. Second, this research was premised around the cultural attitudes of firearms of the home countries of various immigrant suicide decedents. However, when analyzing these decedents, other crucial demographic factors were not considered regarding their culture. Because of this, other exogenous factors, such as the length of exposure to the original culture, the maintenance of the original culture after immigration to the United States, and the length of exposure to US culture, will need to be considered in future research. Third, we were unable to examine the physical availability of or accessibility to firearms at the regional level in the United States due to a lack of relevant data, such as the firearm possession rate at the state level. Fourth, the findings of this research cannot be generalized to the entire country, because the 2017 NVDRS, which contains the most recent scientific data that include detailed information about suicide, obtained data from only 37 states. Fifth, the foreign-born suicide decedents in this research include immigrants with diverse citizenship/residency statuses, including permanent residents, temporary workers, students, visitors, and illegal immigrants. This indicates that their use of a means of suicide may have been impacted by other extenuating factors, such as practical barriers to purchasing guns, rather than cultural familiarity. Sixth, this cross-national comparative study did not consider state-specific differences in the United States, because national-level data were analyzed. Further studies on suicide by firearms in the United States will benefit from considering state-specific gun policies or contexts. Lastly, there was a lack of data on statistics that internationally compare suicide trends by country with regard to US- and foreign-born residents of the country and their suicide rates. This study used the firearm possession rates in the home countries of suicide decedents to indirectly identify potential associations. If such statistics exist and are used in future studies, more direct and robust implications can be drawn.

### Conclusion

Despite limitations, this research has contributed to expanding our understanding of how cultural influences affect higher firearm suicide rates in the United States. Our research shows that nativity is related to firearm suicide rates. Those from countries with high firearm possession rates show a similar propensity for firearm suicide as US-born Americans. The firearm suicide rate is significantly lower among immigrants from countries with a low firearm possession rate. Based on these findings, it is arguable that the high prevalence of firearm possession in the United States contributes to the country’s high firearm suicide rate by promoting public familiarity toward firearms. Consequentially, US-born individuals are desensitized to gun violence, including suicide by firearms. This is based on the finding that foreign-born individuals from countries with low firearm possession rates have a lower tendency to use firearms to die by suicide compared to US-born individuals. However, immigrants from countries with high firearm possession rates are more likely to die by firearm suicide, which supports the association between cultural norms surrounding firearms and the likelihood of dying by firearm suicide [24]. This study has furthered our understanding of the cultural background that contributes to the process by which individuals choose their means of suicide. We hope that this research will contribute to an effective reduction in firearm suicide in the United States as well as improve suicide prevention efforts throughout the country.

## Abbreviations

AOR: adjusted odds ratio
CCA: complete case analysis
CDC: Centers for Disease Control and Prevention
NVDRS: National Violent Death Reporting System
SAS: Small Arms Survey

## Data Availability

The data used in this analysis are available upon request from the Centers for Disease Control and Prevention (CDC) through its restricted-access data process. Our use of these restricted-access National Violent Death Reporting System (NVDRS) data is governed by a data use agreement (DUA) with the CDC. This DUA legally prohibits us from sharing these data with outside investigators. Any investigator can gain access to these restricted-access NVDRS data by contacting the CDC and following the procedures outlined [33].
